# *Brucella* spp. Omp25 Promotes Proteasome-Mediated cGAS Degradation to Attenuate IFN-β Production

**DOI:** 10.3389/fmicb.2021.702881

**Published:** 2021-07-29

**Authors:** Ruizhen Li, Wenli Liu, Xiangrui Yin, Fangfang Zheng, Zhenyu Wang, Xingchen Wu, Xiaohua Zhang, Qian Du, Yong Huang, Dewen Tong

**Affiliations:** ^1^College of Veterinary Medicine, Northwest A&F University, Yangling, China; ^2^School Hospital, Northwest A&F University, Yangling, China

**Keywords:** *Brucella* spp., Omp25, macrophage, cyclic guanosine monophosphate–adenosine monophosphate synthase, interferon β

## Abstract

Type I interferons (IFN), a family of cytokines widely expressed in various tissues, play important roles in anti-infection immunity. Nevertheless, it is not known whether *Brucella* spp. could interfere with IFN-I production induced by other pathogens. This study investigated the regulatory roles of *Brucella* outer membrane protein (Omp)25 on the IFN-I signaling pathway and found that Omp25 inhibited the production of IFN-β and its downstream IFN-stimulated genes induced by various DNA viruses or IFN-stimulatory DNA in human, murine, porcine, bovine, and ovine monocyte/macrophages or peripheral blood mononuclear cells. *Brucella* Omp25 suppressed the phosphorylation of stimulator of IFN genes (STINGs) and IFN regulatory factor 3 and nuclear translocation of phosphorylated IFN regulatory factor 3 in pseudorabies virus- or herpes simplex virus-1-infected murine, human, or porcine macrophages. Furthermore, we found that *Brucella* Omp25 promoted cyclic guanosine monophosphate–adenosine monophosphate synthase (cGAS) degradation *via* the proteasome-dependent pathway, resulting in a decreased cyclic guanosine monophosphate–adenosine monophosphate production and downstream signaling activation upon DNA virus infection or IFN-stimulatory DNA stimulation. Mapping the predominant function domain of Omp25 showed that the amino acids 161 to 184 of Omp25 were required for Omp25-induced cGAS degradation, among which five amino acid residues (R176, Y179, R180, Y181, and Y184) were required for the inhibitory effect of Omp25 on IFN-β induction. Altogether, our results demonstrated that *Brucella* Omp25 inhibits cGAS STING signaling pathway-induced IFN-β *via* facilitating the ubiquitin–proteasome-dependent degradation of cGAS in various mammalian monocyte/macrophages.

## Introduction

*Brucella* spp. is a gram-negative facultative intracellular bacterium that causes brucellosis with clinical manifestations of arthritis, endocarditis, meningitis, abortion, and infertility (particularly in cattle) in humans and animals, resulting in significant economic losses to the livestock industry (Baldi and Giambartolomei, 2013a, b; de Figueiredo et al., 2015). *Brucella* has the propensity to localize inside macrophages, where it inhibits innate immune responses (Campos et al., 2014). The major outer membrane proteins (Omps) of *Brucella* spp. have been identified as the predominant virulence factors in promoting *Brucella* spp. infection and pathogenicity and the coinfection with other pathogens (Machelart et al., 2018; Pasquevich et al., 2019). Previous studies have shown that Omp25 plays a critical role in suppressing tumor necrosis factor-alpha (TNF-α) and interleukin (IL)-12 expression induced by other pathogens (Jubier-Maurin et al., 2001; Cui et al., 2017; Luo et al., 2017). However, it is unknown if *Brucella* infection might increase other pathogen infections by interfering with the induction of type I interferon (IFN) and the function of major Omps.

According to their apparent molecular mass, the Omps were classified into 36- to 38-kDa OMPs or group 2 porin proteins, 25- to 27-kDa, and 31- to 34-kDa OMPs, which belong to group 3 proteins (Cloeckaert et al., 2002). Omp10 is a kind of outer membrane lipoprotein belonging to 31- to 34-kDa Omps, which is necessary for *Brucella* spp. 544 virulence (Tibor et al., 2002). Omp2a is one of the porin proteins belonging to 36- to 38-kDa Omps (Cloeckaert et al., 2002). Omp25, an outer membrane protein of *Brucella*, belonging to the group 3 proteins (Guzman-Verri et al., 2002; Vizcaino et al., 2004), is a *Brucella* key virulence factor (Glowacka et al., 2018) and is involved in the negative regulation of TNF-α production during infection of human macrophages (Jubier-Maurin et al., 2001). In addition, Omp25 is often regulated by environmental signals and plays an important role in bacterial pathogenesis by enhancing the adaptability to various environments (Caro-Hernandez et al., 2007). Our previous studies have demonstrated that *Brucella suis* Omp25 downregulates the production of TNF-α *via* regulation of different microRNAs to promote intracellular survival in porcine and murine macrophages (Luo et al., 2017) and negatively regulates IL-12 production at both transcriptional and post-transcriptional levels through regulation of programmed cell death protein 1 signaling by inducing miR-155, -23b, and -21-5p (Cui et al., 2017). However, the role of Omp25 in regulating the production of IFN-β has not been defined.

The present study investigated the roles of Omp25 in the regulation of DNA viruses or IFN-stimulatory DNA (ISD)-induced IFN-β expression. We found that Omp25 effectively inhibited the cyclic guanosine monophosphate–adenosine monophosphate (cGAMP) synthase (cGAS)–stimulator of IFN genes (STINGs)-mediated induction of IFN-β and downstream IFN-stimulated genes (ISGs) upon DNA viruses or ISD stimulation by promoting the degradation of cGAS. Furthermore, we determined the specific domains of Omp25 responsible for cGAS degradation by reverse transcription-quantitative polymerase chain reaction (qRT-PCR) or Western blotting for quantitative measurement of messenger RNA (mRNA) and protein levels and its regulation of the IFN-I signaling pathway.

## Materials and Methods

### Sample Collections

Blood samples were collected from 105 bovines from China’s northwest district, including three provinces (Shanxi, Ningxia, and Xinjiang), and most samples were collected from bovines with obvious clinical symptoms (such as abortion, conjunctivitis, and rhinotracheitis). Some samples were collected from clinically healthy animals by conventional detection. Whole blood samples were collected into ethylenediaminetetraacetic acid (EDTA)-coated tubes and stored at −20°C until DNA isolation. Blood samples were collected into tubes without an anticoagulant for separation of serum, allowing to clot at room temperature, and centrifuged at 2,000 rpm/min for 10 min. Serum was collected and kept at −80°C. Other clinically common viral infections (such as bovine viral diarrhea virus, parainfluenza virus, and bovine papillomavirus) were excluded from *Brucella*-positive samples by enzyme-linked immunosorbent assay (ELISA). According to the manufacturer’s instructions, commercial ELISA kits were used to detect the antibodies against *Brucella* spp. (F751174-A, Fankewei, Shanghai, China), bovine herpesvirus (BoHV)-1 (F75165-A, Fankewei, Shanghai, China), BoHV-4 (F75169-A, Fankewei, Shanghai, China), bovine viral diarrhea virus (F6732-A, Fankewei, Shanghai, China), parainfluenza virus (F75170-A, Fankewei, Shanghai, China), and bovine papillomavirus (F75022-A, Fankewei, Shanghai, China). All operations were carried out at the biosafety level 2 laboratory.

### Cell Culture

Porcine alveolar macrophages (3D4/21 cells), mouse macrophage cell line (RAW264.7 cells), human monocytic cell line THP-1, Vero cells, Madin–Darby bovine kidney cell line (MDBK), and human embryonic kidney 293 T cell line (HEK-293T cells) were all purchased from American Type Tissue Culture (Manassas, VA, United States) and stored in our laboratory. RAW264.7 cells, Vero cells, MDBK cells, and HEK-293T cells were cultured in Dulbecco’s modified Eagle’s medium (DMEM) supplemented with 10% heat-inactivated fetal bovine serum (FBS) (Hyclone, Thermo Scientific HyClone, Beijing, China). 3D4/21 and THP-1 cells were maintained in Roswell Park Memorial Institute-1640 medium (Invitrogen, Carlsbad, CA, United States) with 10% heat-inactivated FBS, sodium pyruvate, non-essential amino acids. All cells were cultured in a fully humidified atmosphere containing 5% carbon dioxide at 37°C.

### Viruses and Bacteria Strains

Porcine parvovirus (PPV China-XY) (Genbank: MK993540) was isolated and stored in our laboratory. Pseudorabies virus (PRV GD0304) (Genbank: MH582511.1), herpes simplex virus-1 (HSV-1) (Genbank: KC140233.1), BoHV-1 (NM2_2016) (Genbank: MT179818.1), and BoHV-4 J4034 (Genbank: MN735177.1) were stored in our laboratory.

*Brucella abortus* (Genbank: X79284.1), *B. suis* (Genbank: U39397.1), *Brucella melitensis* (Genbank: JF918759.1), *Brucella canis* (Genbank: U39358.1), *Brucella ovis* (Genbank: U33004.1), and *Brucella neotomae* (Genbank: U39359.1) were also stored in our laboratory.

### Cell Isolation and Culture

Peripheral blood mononuclear cells (PBMCs) were prepared from fresh-heparinized venous blood of bovine and ovine by centrifugation over Ficoll-Histopaque (Sigma, St. Louis, MO, United States) as described (Huang et al., 2011). M/MΦs cells were enriched from PBMCs using Ficoll-Percoll gradients (GE Healthcare) and further purified by anti-CD14 magnetic beads with column purification according to the manufacturer’s instructions (Miltenyi Biotec; purity of cells >95%). The enriched cells were cultured in Roswell Park Memorial Institute-1640 medium supplemented with 10% FBS.

### Lentivirus Production

The full-length omp25 (Genbank: X79284.1), omp10 (Genbank: L27995.1), and omp2a (Genbank: AY008719.1) were synthesized and cloned to T vector by Sangon Biotech. The omp25 (Δ161-184) and omp25-5M were amplified by overlap PCR, all sequences were confirmed by sequencing analysis (Sangon Biotech, Shanghai, China), and then they were all cloned into PCDH-CMV-MCS-EF1-copGFP (Addgene, Cambridge, MA, United States) vector according to the manufacturer’s instructions in our laboratory, which were then co-transfected with psPAX2 and PMD2-VSV (Addgene, Cambridge, MA, United States) plasmids into HEK-293T cells for 48 h to generate individual lentivirus particles. Supernatants containing lentiviral particles were harvested and concentrated, and viral titer was determined through infecting HEK-293T cells (Sastry et al., 2002).

### Enzyme-Linked Immunosorbent Assay

3D4/21, THP-1, and RAW264.7 cells that adhered to six-well plates were infected with 100 multiplicity of infections (MOIs) of lentivirus infection for 48 h and then infected with HSV-1 (10 MOI) or transfected with ISD (2 μg/ml) for another 24 h. According to the manufacturer’s instructions, supernatants were harvested to measure IFN-β secretion by ELISA kit (R&D, Minneapolis, MN, United States).

### RNA Extraction and Reverse Transcription-Quantitative Polymerase Chain Reaction

3D4/21, THP-1, and RAW264.7 cells that adhered to six-well plates were infected with 100 MOIs of lentivirus infection for 48 h and then infected with HSV-1 (10 MOI) or transfected with ISD (2 μg/ml) for another 6 h. Bovine PBMCs and ovine PBMCs that adhered to six-well plates were infected with 100 MOIs of lentivirus infection for 48 h, then infected with BoHV-1 or transfected with ISD (2 μg/ml) for another 6 h. IFN-β, ISG56, and C-X-C motif chemokine ligand 10 (IP-10) mRNAs were quantified by qRT-PCR. The primer sequences for real-time PCR are shown in Table 1. According to the manufacturer’s protocol, the total cellular RNA was isolated by TRIZOL. RNA concentration and purity were measured using a NanoDrop spectrophotometer (Thermo Fisher Scientific, Waltham, MA, United States), and equal amounts of RNA (1 μg) were used for qRT-PCR analysis. Relative mRNA levels were detected by an Applied Biosystems QuantStudio 6&7 (Applied Biosystems, Grand Island, NY, United States) and calculated using β-actin as endogenous control, respectively, following the 2^–Δ^ ^Δ^ ^*Ct*^ method.

**TABLE 1:** Primers in this study.

**Primer name**	**Forward primer**	**Reverse primer**
mu IFNβ	ATGAACTCCACCAGCAGACAG	ACCACCATCCAGGCGTAGC
mu ISG56	CGTAGCCTATCGCCAAGATTTA	AGCTTTAGGGCAAGGAGAAC
mu CXCL-10	GCCGTCATTTTCTGCCTC	CGTCCTTGCGAGAGGGAT
mu β-actin	GGCTATGCTCTCCCTCACG	CGCTCGGTCAGGATCTTCAT
hu IFNβ	AGGACAGGATGAACTTTGAC	TGATAGACATTAGCCAGGAG
hu ISG56	CCAGAAATAGACTGTGAGGAAGG	CCCTATCTGGTGATGCAGTAAG
hu CXCL-10	TGGCATTCAAGGAGTACCTC	TTGTAGCAATGATCTCAACACG
hu β-actin	GACCACCTTCAACTCCATCAT	CCTGCTTGCTAATCCACATCT
sus IFNβ	AACCACCACAATTCCAGAGGG	GGTTTCATTCCAGCCAGTGC
sus ISG56	TCAGAGGTGAGAAGGCTGGT	GCTTCCTGCAAGTGTCCTTC
sus CXCL-10	TTCGCTGTACCTGCATCAAG	CAACATGTGGGCAAGATTGA
sus β-actin	GGACTTCGAGCAGGAGATGG	AGGAAGGAGGGCTGGAAGAG
bovine IFNβ	CCTGTGCCTGATTTCATCATGA	AAAGAGCTGTGGTGGAGAAACAC
bovine ISG56	GAATTATGAACGGGCCAAAG	CCCTCCAGGCGATAGACA
bovine CXCL-10	TTCAGGCAGTCTGAGCCTAC	ACGTGGGCAGGATTGACTTG
bovine β-actin	GATGAGATTGGCATGGCTTTA	AACCGACTGCTGTCACCTTC
ovine IFNβ	AATCGTCTGGAGCCAATCTG	GATGTTCAGTCACGGAGGT
ovine ISG56	TGGCAGAAATGTACGCAGAAGC	AAATCGGCCGTAGTGGAAATGTAT
ovine CXCL-10	CACTCCTCAACTCTTCAGGC	CCATTCCTTTTCATTGTGGC
ovine β-actin	AGAGCTACGAGCTGCCTGAC	AGCACTGTGTTGGCGTACAG
BHV-1gB	AAGCGCAAAAACGTGTG	TGCAGGTACAGCTTGGC
BHV-4gB	CCCTTCTTTACCACCACCTACA	TGCCATAGCAGAGAAACAATGA
1-50	CCGGAATTCATGGATTACAAGGATGACGA-CGATAAGCGCACTCTTAAGTCTCTCGTAA	CGCGGATCCTTAGGTATAGCCAC-CAGCCCAGCTA
51-213	CCGGAATTCATGGATTACAAGGATGACGA-CGATAAGGGTCTTTACCTTGGCTAT	CGCGGATCCTTAGAACTTGTAG-CCGATGCCGACG
1-107	CCGGAATTCATGGATTACAAGGATGACGA-C GATAAGCGCACTCTTAAGTCTCTCG	CGCGGATCCTTAGTCCTTGGA-CTTCTTGGCCCAG
108-213	CCGGAATTCATGGATTACAAGGATGACGA-CGA TAAGGGCCTGGAAGTCAAGCAGG	CGCGGATCCTTAGAACTTGTA-GCCGATGCCGACG
1-133	CCGGAATTCATGGATTACAAGGATGACGA-CGATAAGCGCACTCTTAAGTCTCTCG	CGCGGATCCTTAGTACGGCATA-ACCGGGTTCAGG
134-213	CCGGAATTCATGGATTACAAGGATGACGA-CGATAAGCTCACGGCTGGTATTGC	CGCGGATCCTTAGAACTTGTAG-CCGATGCCGACG
1-160	CCGGAATTCATGGATTACAAGGATGACGA-CGATAAGCGCACTCTTAAGTCTCTCG	CGCGGATCCTTACGTCCAACCC-ACGCGGAACTTG
161-213	CCGGAATTCATGGATTACAAGGATGACGAC-GATA AGGGGTTGGACGGCTGGTGC	CGCGGATCCTTAGAACTTGTAGC-CGATGCCGACG
1-184	CCGGAATTCATGGATTACAAGGATGACG-ACGA TAAGCGCACTCTTAAGTCTCTCG	CGCGGATCCTTAGTACTGGGTGT-AACGGTACTCA
185-213	CCGGAATTCATGGATTACAAGGATGACG-AC GATAAGGGCAACAAGAACTATGAT	CGCGGATCCTTAGAACTTGTAGC-CGATGCCGACG
Omp25-△161-184)-2	GGCAACAAGAACTATGATCTGGCCGG	CGCGGATCCTTAGAACTTGTAGCCGATGCC
Omp25-△161-184)-1	CCGGAATTCATGGATTACAAGGATGAC-GACGATAAGCGCACTCTTAAGTCTCTC	CATAGTTCTTGTTGCCCGTCCAACCCAC-GCGGAACTTGCTT
Omp25	CCGGAATTCATGGATTACAAGGATGAC-GACGATAAGCGCACTCTTAAGTCTCTC	CGCGGATCCTTAGAACTTGTAG-CCGATGCC
Omp10	CCGGAATTCATGGATTACAAGGATGACG ACGATAAGGAGAGCATGGACATGAAACG	CGCGGATCCTCAGCCGGCGTT GCGGCGGGTGAGC
Omp2a	GCTCTAGAATGGATTACAAGGATGACGAC GATAAGAACATCAAGAGCCTTCTCC	CGCGGATCCTTAGAACGAGCG CTGGAAGCGAACG
Omp25-5M1	CCGGAATTCATGGATTACAAGGATGAC-GACGATAAGCGCACTCTTAAGTCTCTC	GGTTGCGGCAGCCTCAACTGCGCC-CAGGATGTTGT
Omp25-5M2	GAGGCTGCCGCAACCCAGGCTGG-CAACAAGAACTATGATCTGGCCG	CGCGGATCCTTAGAACTTGTAGC-CGATGCC

*mus: mus musculus; homo: Homo sapiens; sus: sus scrofa.*

### Western Blotting

The cells cultured in a 100-mm-diameter dish (Thermo Fisher Scientific, Waltham, MA, United States) were infected with 100 MOI of Lv-Omp25 or Lv-GFP for 36 or 48 h, then treated with dimethyl sulfoxide, chloroquine (CQ; 20 μM), or MG132 (5 μM) in the presence of cycloheximide (CHX; 100 μg/ml) for another 12 h or further infected by HSV-1 (10 MOI) or PRV (1 MOI) for 6 h; cells were suspended in radioimmunoprecipitation assay lysis buffer (Thermo Fisher Scientific, Waltham, MA, United States) supplemented with protease inhibitor (Sigma Aldrich, St. Louis, MO, United States) on ice for 30 min; cytosol and nuclear fractions were isolated according to manufacturer’s instruction (Thermo Fisher Scientific, Waltham, MA, United States). In detail, infected cells were briefly treated with trypsin-EDTA and gently resuspended in DMEM, then centrifuged at 3,000 × *g*/min for 5 min and washed with PBS. After completely aspirating the PBS, 200 μl of nuclear isolation buffer [1.28-M sucrose, 40-mM Tris–hydrochloride (pH 7.5), 20-mM magnesium chloride, and 4% Triton X-100], 200-μl PBS, and 600-μl water were added to each sample, and the pellets were gently resuspended and placed on ice for 20 min. Samples were centrifuged at 2,500/min for 15 min. Supernatants (cytoplasmic fractions) were collected, and then the pellets (nuclear fraction) were gently resuspended in 500-μl 0.1-M PBS. Finally, the protein was eluted by boiling for 10 min in a 2 × loading buffer. Forty-microgram proteins in each lane were subjected to sodium dodecyl sulfate–polyacrylamide gel electrophoresis and transferred to polyvinyl difluoride membranes (Millipore Corp., Atlanta, GA, United States) for Western blotting. After blocking the membrane with 5% non-fat dry milk for 2 h, we incubated it with primary antibodies at 4°C overnight. The cells were treated with ISD (Invivogen tlrl-ISDN, Cayla, France), protein synthesis inhibitor (CHX; Sigma, St. Louis, MO, United States), lysosome inhibitor (CQ; Sigma, St. Louis, MO, United States), and proteasome inhibitors (MG132, Beyotime, Shanghai, China). Primary antibodies included mouse anti-β-actin (A00702; Genscript, Nanjing, China), mouse anti-tubulin (A01410-100; Genscript, Nanjing, China), rabbit anti-cGAS (31659; CST, Danvers, MA, United States), rabbit anti-phospho-STING (72971; 50907; CST, Danvers, MA, United States), rabbit anti-STING (ab181125; Abcam, Cambridge, MA, United Kingdom), rabbit anti-STING (19851-1-AP; Proteintech, Chicago, IL, United States), anti-phospho-IFN regulatory factor 3 (IRF3) (4947; CST, Danvers, MA, United States), rabbit anti-IRF3 (4302; CST, Danvers, MA, United States), mouse anti-HA antibody (H3663; Sigma, St. Louis, MO, United States), rabbit anti-Flag antibody (701629, Invitrogen, Carlsbad, CA), and rabbit anti-histone H3 (4499; CST, Danvers, MA, United States). The anti-cGAS polyclonal antibody was obtained from a mouse immunized with purified full-length cGAS protein expressed by the pET32a vector in *Escherichia coli*. Horseradish peroxidase-conjugated anti-mouse immunoglobulin G (BM2002; Wuhan Boster Biotech, Wuhan, China) or anti-rabbit immunoglobulin G (BA1058; Wuhan Boster Biotech, Wuhan, China) was incubated at room temperature for 1 h. According to the manufacturer’s instructions, enhanced chemiluminescence (Bio-Rad, Hercules, CA, United States) was used for detection.

### Cyclic Guanosine Monophosphate–Adenosine Monophosphate Activity Assay

Infected or transfected cells were trypsinized and washed with PBS. Pelleted cells were resuspended in the indicated volume of PBS andlysed by freezing at −80°C. Cell extracts were nuclease and heat-treated as previously described (Gao et al., 2013) with modifications. Briefly, cell extracts were incubated with ∼1 U/μl Benzonase (Sigma-Aldrich, St. Louis, MO, United States) for 30 min at 37°C. Cell extracts were then heated at 95°C for 5 min and centrifuged for 5 min at maximum speed (∼16,000 × *g*) in an Eppendorf microcentrifuge. RAW264.7, THP-1, and 3D4/21 cells were used as a reporter cell line to measure cGAMP production. Cells were permeabilized as described previously (Woodward et al., 2010) with modifications. Briefly, media was aspirated from RAW264.7, THP-1 or 3D4/21 cells, and digitonin permeabilization solution [50-mM 4-(2-hydroxyethyl)-1-piperazineethanesulfonic acid (pH 7.0), 100-mM potassium chloride, 85-mM sucrose, 3-mM magnesium chloride, 0.2% bovine serum albumin, 1-mM adenosine triphosphate, 0.1-mM dithiothreitol, and 10-μg/ml digitonin] was added with 25 μl per well-treated cell extracts or 2′-3′-cGAMP (Invivogen tlrl-cGAS, Cayla, France) as a positive control. RAW264.7, THP-1, and 3D4/21 reporter cells were incubated with extracts for 30 min at 37°C and then replaced with supplemented media. RNA was harvested 6 h after the initial addition of extracts, and qRT-PCR was performed, as described earlier.

### Immunoprecipitation Assay

According to the manufacturer’s instructions, cells were cultured in a 100-mm diameter dish and co-transfected with indicated plasmids using the Lipofectamine 3000 reagent (Invitrogen, Carlsbad, CA, United States); 24 h later, the cells were infected with 100 MOI of Lv-Omp25 or Lv-GFP. At 36-h post-infection, cells were treated with MG132 (5 μM) for another 12 h, then the cells lysed with lysis buffer [150-mM sodium chloride, 50-mM Tris–hydrochloride (pH 7.4), 1% Nonidet P-40, 0.5% Triton X-100, 1-mM EDTA, 0.1% sodium deoxycholate, 1-mM dithiothreitol, 0.2-mM phenylmethylsulfonyl fluoride, and protease inhibitor protease inhibitor cocktail (Sigma-Aldrich, St. Louis, MO, United States)] on ice for 30 min. The cell lysate supernatant was collected by centrifuging for 15 min at 13,000 × *g* and incubated with protein A-agarose (Santa Cruz, California, CA, United States) for pre-cleaning of non-specific proteins for 1 h at 4°C. The samples were centrifuged, and the supernatant was incubated with the indicated antibodies overnight at 4°C. The complex was incubated with protein A-agarose again for 30 min at room temperature and then centrifuged at 2,000 × *g* for 10 s and washed three times with PBS. Finally, the bound proteins were eluted by boiling for 10 min in 2 × loading buffer and analyzed by Western blotting using the indicated antibodies.

### Herpes Simplex Virus 1 and Pseudorabies Virus Plaque Assay

Vero cells were seeded into six-well plates at a density of 2 × 10^6^ viable cells per well. On the following day, cells were infected with 100 MOI per cell of Lv-Omp25 or Lv-GFP. At 48 h after infection, cells were incubated with HSV-1 (10 MOI) or PRV (1 MOI) for 2 h at room temperature on a rocking apparatus, then aspirated the inoculum and washed with PBS three times, overlayed the cells with 2 ml of 2 × DMEM supplemented with 2% methylcellulose, 2% FBS per well. Plates were incubated at 37°C and 5% carbon dioxide for 3 days. Cells were stained with 1-ml 1% crystal violet hydrate solution containing 0.6% sodium chloride and 2% formaldehyde per well for 30 min to visualize plaques.

### Ethics Statement

This research was approved by the Institutional Animal Care and Use Committee of Northwest A&F University and was performed according to the Animal Ethics Procedures and Guidelines of the People’s Republic of China. No other specific permissions were required for these activities. This study did not involve endangered or protected species.

### Statistical Analysis

The results are representative of three independent experiments. The data are presented as mean ± standard error of the mean (standard deviation). Comparisons between the two groups were performed by unpaired Student *t*-test, whereas multiple group data were analyzed by analysis of variance, followed by Bonferroni *post hoc* test. Statistically significant and very significant results were defined as *P* < 0.05 and *P* < 0.01.

## Results

### *Brucella* spp.-Positive Cattle Are More Susceptible to Bovine Herpes Viruses-1 and -4

**FIGURE 1:**
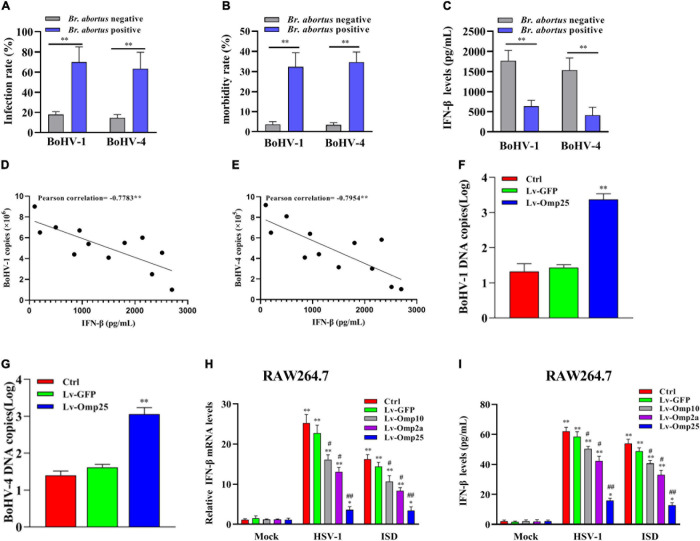
*Brucella* spp.-positive cattle are more susceptible to BoHV-1 and BoHV-4. **(A,B)** Serum were collected from *Brucella* spp.-positive or -negative cattle, and all samples were detected with specific ELISA kits. **P < 0.01 versus *Brucella* spp.-negative group. **(C)** IFN-β levels were lower in *Brucella* spp.-positive cattle than that in *Brucella* spp.-negative cattle. Serum was collected from *Brucella* spp.-positive or -negative cattle coinfection BoHV-1 or BoHV-4; level of IFN-β was detected with ELISA kit. **P < 0.01 versus *Brucella* spp.-negative group. **(D,E)** Level of IFN-β is inversely correlated with either BoHV-1 or BoHV-4 copies in these cattle that infected with or without *Brucella* spp. ELISA was applied to detect production of IFN-β in different cattle infected with BoHV-1 or BoHV-4 virus; qRT-PCR was applied to detect copies of BoHV-1 or BoHV-4 in these cattle. **P < 0.01 demonstrated that level of IFN-β is inversely correlated with BoHV-1 or BoHV-4 copies. **(F,G)** *Brucella* spp. Omp25 inhibits replication of BoHV-1 and BoHV-4 in MDBK cells. MDBK cells were infected with 100 MOI of Lv-Omp25 or Lv-GFP for 48 h and then infected with BoHV-1 (1 MOI) or BoHV-4 (1 MOI) for another 6 h, qRT-PCR was applied to detect copies of BoHV-1 or BoHV-4. **(H,I)** *Brucella* spp. Omp25 is key protein to inhibit expression of IFN-β induced by HSV-1 or ISD in RAW264.7. RAW264.7s were infected with 100 multiplicity of infection (MOI) of Lv-Omp25, Lv-Omp10, Lv-Omp2a, or Lv-GFP for 48 h and then infected with HSV-1 (10 MOI) or transfected with ISD (2 μg/ml) for another 6 or 24 h. Cells were harvested and subjected to qRT-PCR analysis **(H)** or ELISA analysis **(I)**. *P < 0.05, **P < 0.01 versus mock group, ^#^*P* < 0.05, ^##^*P* < 0.01 versus control or Lv-GFP-infected cells in same secondary or infection stimulation groups. Results are means ± SEMs of three independent experiments.

Firstly, we detected the infection rate and morbidity rate of BoHV-1 and BoHV-4 in *Brucella* spp.-positive and -negative samples. The results showed that the infection and morbidity rates were higher in *Brucella* spp.-positive cattle than *Brucella* spp.-negative cattle (Figures 1A,B). Then, we tested the levels of IFN-β in *Brucella* spp.-positive and -negative cattle that co-infected with BoHV-1 or BoHV-4, and we found that IFN-β levels were lower in *Brucella* spp.-positive cattle than that in *Brucella* spp.-negative cattle (Figure 1C). To further confirm the relationships between IFN-β level and BoHV-1 and BoHV-4 viral load, ELISA was applied to detect the production of IFN-β in different cattle infected with BoHV-1 or BoHV-4; qRT-PCR was applied to detect the copies of BoHV-1 or BoHV-4 in these cattle. The result showed that the level of IFN-β was inversely correlated with BoHV-1 or BoHV-4 copies in these cattle that infected with or without *Brucella* spp. (Figures 1D,E). Then, we detected replication of BoHV-1 and BoHV-4 in MDBK cells with or without infected Lv-Omp25. MDBK cells were infected with 100 MOI of Lv-Omp25 or Lv-GFP for 48 h and then infected with BoHV-1 (1 MOI) or BoHV-4 (1 MOI) for another 6 h; we found that *Brucella* spp. Omp25 inhibited BoHV-1 or BoHV-4 copies in MDBK cells (Figures 1F,G). These data suggested that *Brucella* spp. infection could inhibit IFN-β to promote BoHV-1 or BoHV-4 infection.

Next, we evaluated the effects of three outer membrane proteins of *Brucella* spp. (Omp25, Omp10, and Omp2a) on IFN-β production. RAW264.7 were infected with 100 MOI of Lv-Omp25, Lv-Omp10, Lv-Omp2a, or Lv-GFP for 48 h and then infected with HSV-1 (10 MOI) or transfected with ISD (2 μg/ml) for another 6 or 24 h followed by qRT-PCR detection of IFN-β mRNA levels and ELISA detection of IFN-β secretion levels. We found that Omp25, Omp10, and Omp2a could inhibit IFN-β production, but Omp25 showed a most significant inhibitory effect (Figures 1H,I). Thus, we explored how Omp25 inhibits IFN-β production induced by the DNA virus in this study.

### Omp25 Inhibits DNA Virus and Interferon-Stimulatory DNA-Induced Interferon-β Production in Various Mammalian Monocyte/Macrophages

**FIGURE 2:**
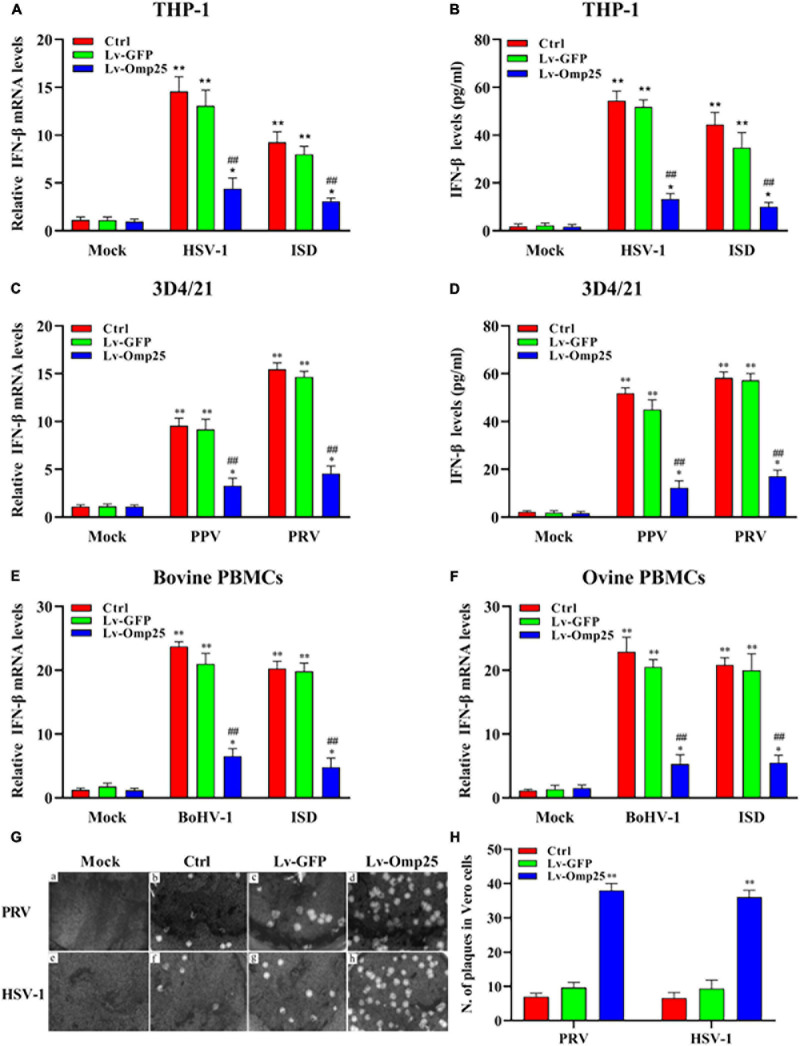
Omp25 inhibits DNA virus and ISD-induced IFN-β expression in various mammalian monocyte/macrophages. **(A)** Omp25 inhibits IFN-β expression in THP-1 cells. THP-1 cells were infected with 100 multiplicity of infection (MOI) of Lv-Omp25 or Lv-GFP for 48 h and then infected with HSV-1 (10 MOI) or transfected with ISD (2 μg/ml) for another 6 h. Cells were harvested and subjected to qRT-PCR analysis. **(B)** Omp25 inhibits IFN-β production in THP-1 cells. THP-1 cells were infected with 100 MOI of Lv-Omp25 or Lv-GFP for 48 h and then infected with HSV-1 (10 MOI) or transfected with ISD (2 μg/ml) for another 24 h. Cells were harvested and subjected to ELISA analysis. **(C,D)** Omp25 inhibits PPV- or PRV-induced IFN-β transcription and expression in 3D4/21 cells. Cells were infected and treated as discussed earlier and then infected with PPV (1 MOI) or PRV (1 MOI) for another 6 or 24 h. Cells were harvested and subjected to qRT-PCR analysis or ELISA analysis. **(E,F)** Omp25 inhibits BoHV-1-induced IFN-β transcription in bovine and ovine PBMCs. Cells were infected and treated as in discussed earlier and then infected with BoHV-1 (1 MOI) or stimulated with ISD (2 μg/ml) for another 6 h. Cells were harvested and subjected to qRT-PCR analysis. **(G,H)** Omp25 inhibits PRV and HSV-1 replication. Vero cells were infected with 100 MOI of Lv-Omp25 or Lv-GFP for 48 h and then infected with HSV-1 (10 MOI) or PRV (1 MOI) for 1 h and overlayed with methylcellulose for 3 days, and then, cells were stained with 5% crystal violet hydrate solution for 30 min **(G)**. Number of plaque by taking average number of viruses in per well **(H)**. Results are means ± SEMs of three independent experiments. *P < 0.05, **P < 0.01 versus mock group, ^#^*P* < 0.05, ^##^*P* < 0.01 versus control or Lv-GFP-infected cells in same secondary infection or stimulation groups.

To investigate the direct effects of Omp25 on IFN-β production in monocyte cells or macrophages, we examined and compared the IFN-β mRNA and protein levels between various Lv-Omp25-infected and Lv-GFP-infected mammalian monocyte/macrophage cells. Upon DNA viruses [HSV-1 (10 MOI), PPV (1 MOI), PRV (1 MOI), and BoHV-1 (1 MOI)] infection or ISD (2 μg/ml) stimulation, IFN-βs were significantly induced in both mRNA and protein levels in THP-1, 3D4/21 cells, bovine PBMCs, and ovine PBMCs (Figures 2A–F), but we found that the levels of IFN-β mRNA in Lv-Omp25-infected cells were lower than that in Lv-GFP-infected various mammalian cells and control cells regardless of DNA viruses infection or ISD stimulation (Figures 2A,C,E,F). We also observed that the production of IFN-β induced by DNA viruses or ISD (2 μg/ml) was also lower in Lv-Omp25-infected cells than in Lv-GFP-infected and control cells (Figures 2B,D). Correspondingly, the replication of PRV and HSV-1 were increased in Lv-Omp25-infected cells relative to either Lv-GFP-infected or control cells in the plaque assay (Figures 2G,H). These results indicated that *Brucella* Omp25 could directly inhibit the production of DNA viruses and ISD-induced IFN-β expression at both protein and mRNA levels in various mammalian monocyte/macrophages.

### Outer Membrane Protein 25 Inhibits DNA Viruses and Interferon-Stimulatory DNA-Induced Transcription of Interferon-Stimulated Gene

**FIGURE 3:**
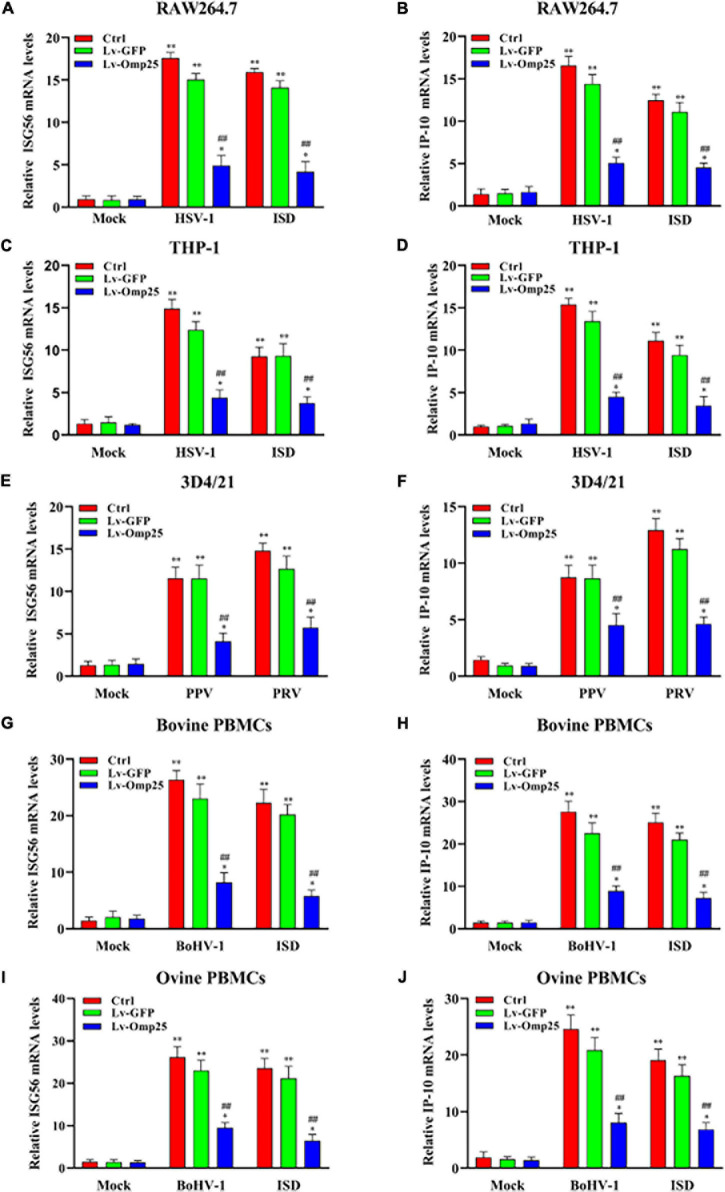
Omp25 inhibits DNA virus and ISD-induced ISG56 and IP-10 expression in various mammalian monocyte/macrophages. Cells were infected with 100 MOI of Lv-Omp25 or Lv-GFP for 48 h and then infected with HSV-1 (10 MOI), PPV (1 MOI), PRV (1 MOI), or BoHV-1 (1 MOI) or transfected with ISD (2 μg/ml) for another 6 h. Cells were harvested and subjected to qRT-PCR analysis to measure ISG56 mRNA levels **(A,C,E,G,I)** or IP-10 mRNA levels **(B,D,F,H,J)**. *P < 0.05, **P < 0.01 versus mock group, ^#^*P* < 0.05, ^##^*P* < 0.01 versus Lv-GFP-infected cells in same groups.

To investigate the effects of Omp25 on transcription of IFN-β-stimulated genes, we analyzed ISG56 and C-X-C motif chemokine ligand 10 (IP-10) expressions by qRT-PCR in various mammalian monocyte/macrophages. Upon DNA virus infection or ISD (2 μg/ml) stimulation, the transcription of ISG56 in cells expressing Omp25 was markedly lower than in Lv-GFP-infected or control cells (Figures 3A,C,E,G,I). Similarly, the transcriptional levels of IP-10 were also lower in Omp25-expressing cells than in Lv-GFP-infected or control cells (Figures 3B,D,F,H,J). Meanwhile, the ISG56 and IP-10 mRNA levels were higher in DNA virus-infected cells or ISD (2 μg/ml)-stimulated cells than in mock treatment (Figures 3A–J). These results demonstrated that Omp25 inhibited DNA viruses and ISD-induced transcription of ISG.

### Outer Membrane Protein 25 Negatively Regulates DNA Viruses-Activated Stimulator of Interferon Genes and Interferon Regulatory Factor 3 Phosphorylation

**FIGURE 4:**
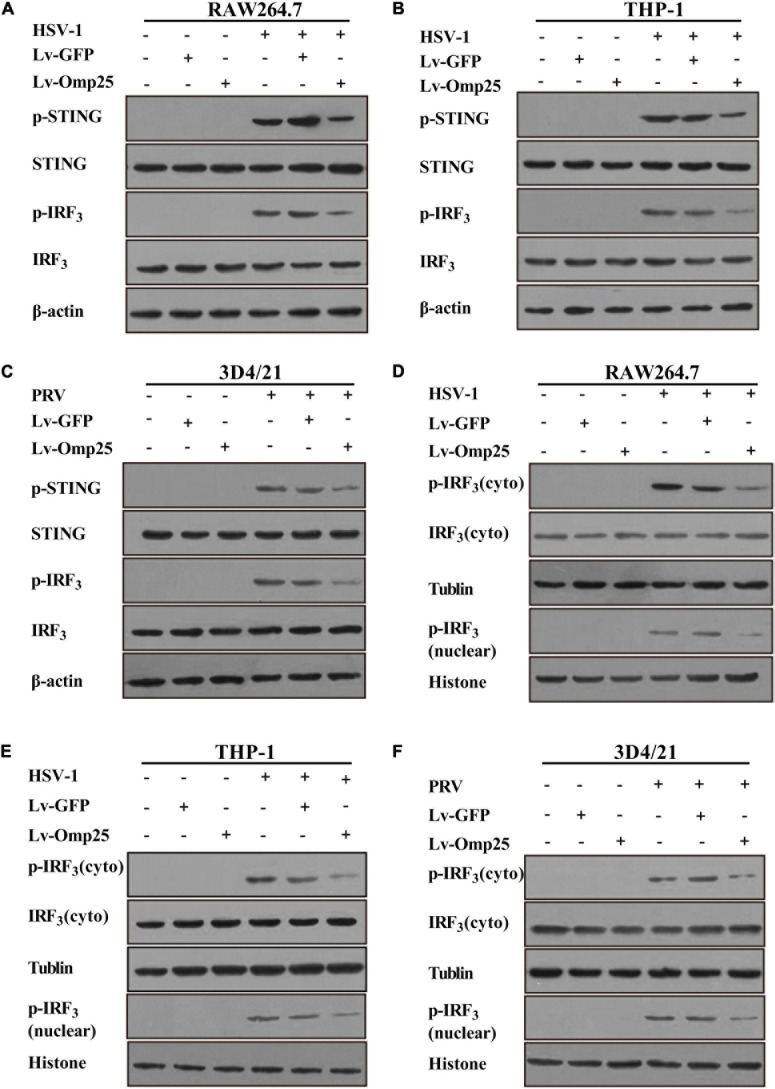
Omp25 inhibits activation of cGAS/STING pathway. **(A–C)** Omp25 inhibits phosphorylation of STING and IRF3 (p-STING, p-IRF3) in murine, human, and porcine macrophages. Cells were infected with 100 MOI of Lv-Omp25 or Lv-GFP for 48 h and then further infected by HSV-1 (10 MOI) or PRV (1 MOI) for 6 h. Cells were harvested and subjected to detect level of p-STING and p-IRF3 by Western blotting. **(D–F)** Omp25 affects distribution of cytoplasmic and nuclear p-IRF3. Cells were infected and treated as in panels **(A–C)**, cytoplasm and nuclear were separated, and level of p-IRF3 in cytoplasm and nuclear were detected by Western blotting.

To further investigate how Omp25 inhibited IFN-I production, we tested phosphorylation of STING and IRF3 in RAW264.7, THP-1, and 3D4/21 cells. The results showed that following HSV-1 or PRV infection, the phosphorylation of STING and IRF3 significantly decreased in cells overexpressing Omp25 compared with that in Lv-GFP-infected or control cells (Figures 4A–C), and also less nuclear p-IRF3 were detected in Lv-Omp25-infected cells compared to that in Lv-GFP-infected or control cells (Figures 4D–F). Thus, these data suggested that Omp25 negatively regulated DNA viruses-activated STING and IRF3 phosphorylation.

### Outer Membrane Protein 25 Dampens Interferon-β Induction by Reducing the Activity of Cyclic Guanosine Monophosphate–Adenosine Monophosphate Synthase

**FIGURE 5:**
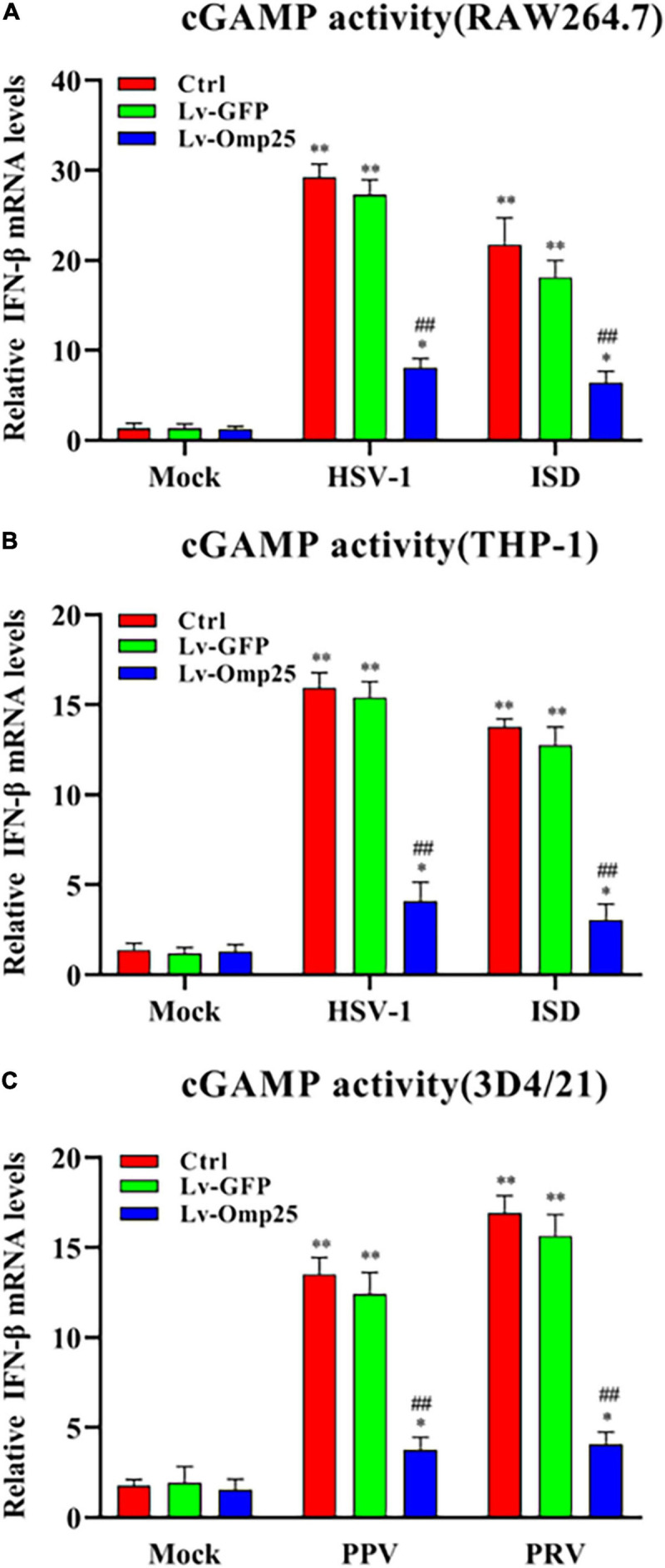
Omp25 inhibits cGAS activity in human, murine, and porcine macrophages. **(A,B)** Omp25 inhibits cGAS activity in RAW264.7 or THP-1 cells. Cells were infected with Lv-Omp25 or Lv-GFP at an MOI of 100 for 48 h, then infected with HSV-1 (10 MOI) or stimulated by ISD (2 μg/ml) another 6 h. Extracts from infected cells were prepared, DNase and heat-treated, and incubated with permeabilized RAW264.7 cells or THP-1 cells for 6 h. IFN-β RNA induction was analyzed by qRT-PCR and normalized to that of β-actin to assess levels of cGAMP in cell extracts. **(C)** Omp25 inhibits cGAS activity in porcine 3D4/21 cells. Cells were infected and treated as in panels **(A,B)**. Extracts from infected cells were prepared and incubated with permeabilized 3D4/21 cells for 6 h. IFN-β RNA induction was analyzed by qRT-PCR. *P < 0.05, **P < 0.01 versus mock group, ^#^*P* < 0.05, ^##^*P* < 0.01 versus Lv-GFP-infected cells in same groups.

To further define the potential mechanisms of Omp25 in regulating the cGAS-STING signaling pathway, we examined the production of cGAMP in RAW264.7, THP-1, and 3D4/21 cells infected with Lv-GFP or Lv-Omp25 under the DNA virus infection and ISD (2 μg/ml) stimulation. cGAMP activity was measured in cell extracts using a modified bioassay by assessing the induction of IFN-β transcripts in a permeabilized secondary reporter cells (RAW264.7, THP-1, and 3D4/21 cells). As shown in Figures 5A–C, cGAMP activity was significantly decreased in the cells infected with Lv-Omp25 than in the cells infected with Lv-GFP or control. These results indicated that Omp25 dampened IFN-β production by reducing the activity of cGAS.

### Outer Membrane Protein 25 Induces the Ubiquitin–Proteasome-Dependent Cyclic Guanosine Monophosphate–Adenosine Monophosphate Synthase Degradation

**FIGURE 6:**
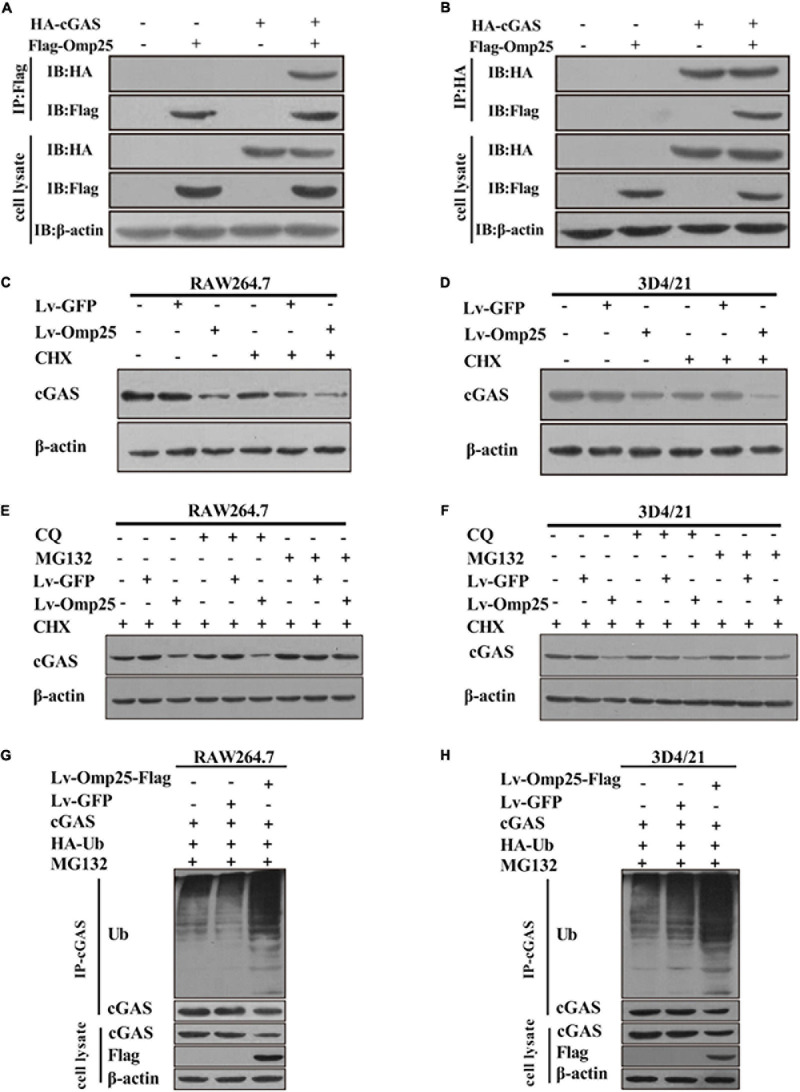
Omp25 reduces level of cGAS protein and induces proteasome-dependent degradation of cGAS. **(A**,**B)** Omp25 interacted with cGAS. Cells were co-expressed with Flag-Omp25 and HA-cGAS for 24 h, and cell lysates were subjected to a co-IP assay using anti-Flag antibody, anti-HA antibody, and anti-β-actin antibody. **(C,D)** Omp25 degraded cGAS in murine and porcine macrophages. RAW264.7 or 3D4/21 cells were infected with 100 MOI of Lv-Omp25 or Lv-GFP for 36 h, then treated with or without CHX (100 μg/ml) for another 12 h. Level of cGAS was detected by Western blotting. **(E,F)** Omp25 promoted degradation of cGAS *via* proteasome-dependent pathway. RAW264.7 or 3D4/21 were infected with 100 MOI of Lv-Omp25 or Lv-GFP for 36 h and treated with dimethyl sulfoxide, CQ (20 μM), or MG132 (5 μM) in presence of CHX for another 12 h, followed by Western blotting to detect levels of cGAS. **(G,H)** Omp25 induced cGAS degradation through ubiquitin–proteasome pathway in RAW264.7 and 3D4/21 cells. Cells were transfected with vector expressing HA-Ub by Lipofectamine 3000 for 24 h, then infected with 100 MOI of Lv-Omp25 or Lv-GFP. At 36-h post-infection, cells were treated with MG132 (5 μM) for another 12 h; ubiquitination level of cGAS protein was determined by Western blotting.

Because Omp25 inhibited cGAS-induced IFN-I production by reducing cGAMP production, we further detected the effects of Omp25 on cGAS protein levels. Firstly, we detected the interaction between Omp25 and cGAS. HEK293T cells were co-transfected with the vector expressing HA-cGAS and Flag-Omp25 for 24 h. The results showed that cGAS interacted with Omp25 (Figures 6A,B). Then, we tested the effect of Omp25 on cGAS in RAW264.7 and 3D4/21 cells. Cells were infected with 100 MOI of Lv-Omp25 or Lv-GFP. At 36-h post-infection, cells were treated with or without protein synthesis inhibitor (CHX, 100 μg/ml), then the level of cGAS protein was determined by Western blotting. The results showed that the protein level of cGAS was significantly reduced in Lv-Omp25-infected cells compared with Lv-GFP-infected or control cells whenever these cells were treated with or without CHX (Figures 6C,D), suggesting that Omp25 can promote cGAS degradation.

Therefore, to clarify which pathway was responsible for the cGAS degradation induced by Omp25 in the proteasome pathway and lysosome pathway, we compared the cGAS degradation in the Lv-Omp25-infected cells treated with either proteasome inhibitor (MG132, 5 μM) or lysosome inhibitor (CQ, 20 μM). Results showed that MG132 treatment could restore the level of cGAS protein in Lv-Omp25-infected cells, similar to the level as that in Lv-GFP-infected or control cells in the presence of CHX, whereas CQ treatment could not (Figures 6E,F). Then, we detected the effect of Omp25 on cGAS ubiquitination in RAW264.7 and 3D4/21 cells. Cells were transfected with the vector expressing HA-Ub for 24 h, then infected with 100 MOI of Lv-Omp25 or Lv-GFP. At 36-h post-infection, cells were treated with MG132 (5 μM) for another 12 h before collecting, then the ubiquitination level of cGAS protein was determined by Western blotting. The results showed that the level of cGAS ubiquitination was markedly increased in Lv-Omp25-infected cells compared with Lv-GFP-infected or control cells (Figures 6G,6H). Altogether, these results suggested that Omp25 induced cGAS degradation through the ubiquitin–proteasome pathway.

### 161 to 184 Amino Acids of Outer Membrane Protein 25 Are Critical for Induction of Cyclic Guanosine Monophosphate–Adenosine Monophosphate Synthase Degradation

**FIGURE 7:**
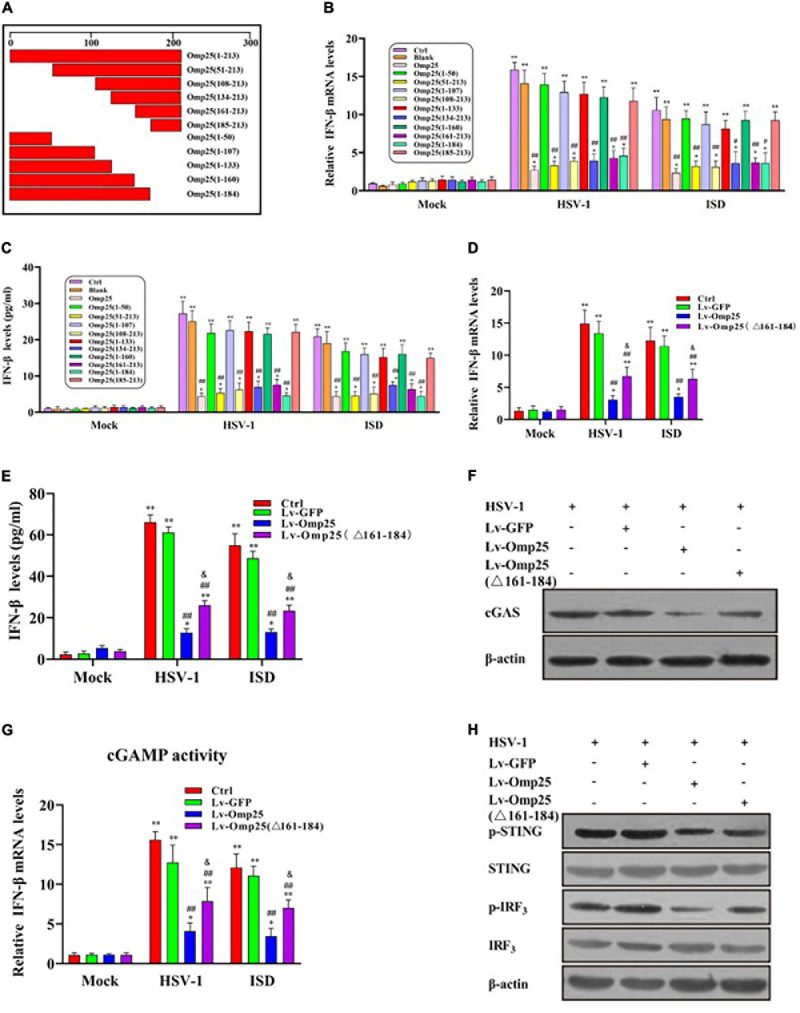
Effect of Omp25 deletion mutants on cGAS in cells. **(A)** Schematic diagram of Omp25 deletion mutants. **(B,C)** Mapping regions of Omp25 that decrease IFN-β mRNA levels or protein levels. RAW264.7 cells were infected with different deletion mutants for 48 h, then infected with HSV-1 (10 MOI) or transfected with ISD (2 μg/ml) for another 6 or 24 h. Cells were harvested and subjected to qRT-PCR analysis or ELISA analysis to examine expression of IFN-β. **(D,E)** Lv-Omp25Δ161–184 showed an attenuated inhibitory effect in transcription and expression of IFN-β. RAW264.7 cells were infected with Lv-Omp25, Lv-Omp25Δ161–184, or Lv-GFP for 48 h, then infected with HSV-1 (10 MOI) or transfected with ISD (2 μg/ml) for another 6 or 24 h. Cells were harvested and subjected to qRT-PCR analysis or ELISA analysis to examine expression of IFN-β. **(F,G)** Omp25Δ161–184 showed a weak inhibitory effect on levels of cGAS and production of cGAMP. Cells were treated as in panels **(D,E)**; protein levels of cGAS and levels of cGAMP in cell extracts were measured by Western blotting or qRT-PCR as described in section “Materials and Methods.” **(H)** Lv-Omp25Δ161–184 showed a weakened inhibitory effect on phosphorylation of STING and IRF3. Cells were treated as in panels **(D,E)**, and phosphorylation of STING and IRF3 were measured by Western blotting. *P < 0.05, **P < 0.01 versus mock group, ^#^*P* < 0.05, ^##^*P* < 0.01 versus Lv-GFP- infected cells in same groups, ^&^*P* < 0.05 versus Lv-Omp25-infected cells in same groups.

To identify the key functional motifs and amino acid residues of Omp25 required to induce cGAS degradation, we constructed 10 deletion mutants of Omp25 (Figure 7A). We examined and compared the inhibitory effects of these Omp25 mutants on the HSV-1 (10 MOI)- or ISD (2 μg/ml)-induced IFN-β expression in mRNA and protein levels. Results showed that Omp25 fragments 51–213, 108–213, 134–213, 161–213, and 1–184 significantly reduced cGAS-induced IFN-β production and full-length Omp25, whereas Omp25 fragments 1–50, 1–107, 1–133, 1–160, and 185–213 failed to exhibit a significant inhibitory effect on cGAS-induced IFN-β production (Figures 7B,C). These results suggested that the fragment (amino acid 161–184) of Omp25 was required to induce cGAS degradation. To further confirm this domain was critical for induction of cGAS degradation, we constructed an Omp25 (161–184 aa) deletion mutant Omp25Δ161-184 and detected the ability of this mutant to induce cGAS degradation. Results showed that Omp25Δ161-184 deletion mutant appeared a significantly attenuated inhibitory ability in transcription and expression of IFN-β induced by HSV-1 infection and ISD stimulation compared with full-length Omp25 but still exhibited a certain inhibitory effect on IFN-β induction relative to Lv-GFP or control (Figures 7D,E). Meanwhile, upon HSV-1 (10 MOI) or ISD (2 μg/ml) stimulation, Omp25Δ161-184 showed a weaken inhibitory effect on the levels of cGAS and the production of cGAMP relative to full-length Omp25 but did decrease cGAS and cGAMP production relative to Lv-GFP infected or control cells (Figures 7F,G). Correspondingly, the phosphorylation of STING and IRF3 was higher in Lv-Omp25Δ161-184-infected cells than that in Lv-Omp25-infected cells but still lower than that in Lv-GFP-infected or control cells (Figure 7H). Taken together, the 161 to 184 amino acids of Omp25 were critical for induction of cGAS degradation and inhibition of IFN-β induction.

### Amino Acid Residues (R176, Y179, R180, Y181, and Y184) Are Critical for Outer Membrane Protein 25 to Inhibit Interferon β Induction

**FIGURE 8:**
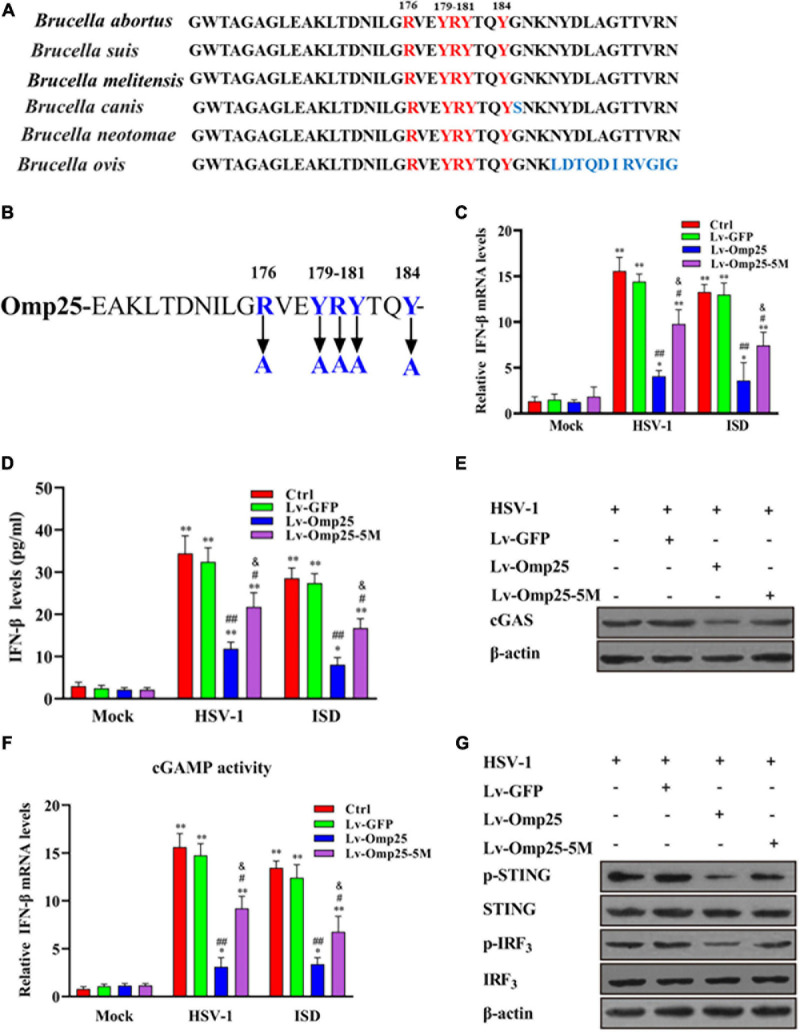
Determination of key amino acid residue in Omp25 plays a critical role in Omp25 inhibition of IFN-β expression. **(A)** Alignment of Omp25 high conserved amino acid motif in different *Brucella* species. **(B)** Schematic diagram of Omp25 mutation in R176, Y179, R180, Y181, and Y184. **(C–G)** RAW264.7 cells were infected with Lv-Omp25, Lv-Omp25-5M, or Lv-GFP for 48 h, then infected with HSV-1 (10 MOI) or transfected with ISD (2 μg/ml) for another 6 or 24 h. Cells were harvested and subjected to qRT-PCR analysis or ELISA analysis to examine expression of IFN-β **(C,D)**; Western blotting was performed to detect levels of cGAS **(E)**; qRT-PCR was used to detect levels of cGAMP in cell extracts as described in section “Materials and Methods” **(F)**, and phosphorylation levels of STING and IRF3 were measured by Western blotting **(G)**. *P < 0.05, **P < 0.01 versus mock group, ^#^*P* < 0.05, ^##^*P* < 0.01 versus Lv-GFP-infected cells in same groups. ^&^*P* < 0.05 versus Lv-Omp25 infected cells in same groups.

Sequence alignment of *Brucella* spp. Omp25 protein revealed that the amino acid residues 158–198 were highly conserved in different *Brucella* species (Figure 8A). Therefore, to identify the key amino acid residues in Omp25 (161–184) that played a critical role in Omp25 inhibition of IFN-β expression, we screened out five amino acid residues (R176, Y179, R180, Y181, and Y184) from the region (aa 158 to 198) (Figure 8B). When these five amino acid residues were replaced by alanine, we observed that the corresponding mutant, Omp25-5M, showed an attenuated inhibitory effect on HSV-1- or ISD-induced IFN-β but remained a certain inhibitory effect on IFN-β induction compared with Lv-GFP-infected or control cells (Figures 8C,D). Furthermore, upon HSV-1 (10 MOI) infection or ISD (2 μg/ml) stimulation, the protein level and activity of cGAS were higher in mutant Omp25-5M-expressing cells than that in wild-type Omp25-expressing cells, although the protein level and activity of cGAS in both Omp25-5M-expressing cells and Omp25-expressing cells were still lower than that in Lv-GFP-infected or control cells (Figures 8E,F). At the same time, the phosphorylation of STING and IRF3 was increased in Omp25-5M-expressing cells compared with Omp25-expressing cells but still lower than that in Lv-GFP-infected or control cells (Figure 8G). Altogether, these results demonstrated that amino acid residues (R176, Y179, R180, Y181, and Y184) were critical for Omp25 to inhibit IFN-β induction.

## Discussion

It is well known that IFN-Is, such as IFN-α and IFN-β, are playing important roles in antiviral and antibacterial infections (Borden et al., 2007; Monroe et al., 2010; McNab et al., 2015). Cheng et al. (2018), Huang B. et al. (2018), and Marinho et al. (2018) have reported that both viral and bacterial intracellular pathogens can induce IFN-I by the cGAS/STING signal pathway. It has been reported that induction of IFN-β by *Mycobacterium tuberculosis* and *Listeria* depends on cytoplasmic cGAS sensing (Hansen et al., 2014; Collins et al., 2015). However, the microbes have developed mechanisms to manipulate this innate immune sensing pathway. For example, some proteins expressed by viruses increase infectivity by interfering with cGAS activation (Wu et al., 2015; Aguirre et al., 2017; Huang Z. F. et al., 2018). In this study, we demonstrated that Omp25 inhibited the expression of IFN-β induced by DNA viruses or ISD *via* degradation of cGAS depending on the ubiquitin–proteasome pathway in mammalian monocyte/macrophages.

Brucellosis, caused by *Brucella*, is a worldwide zoonosis affecting animal and human health, which often causes immunosuppression (Renoux and Renoux, 1977). Forestier et al. (2000) reported that the membrane ingredients of *Brucella* spp. (such as lipopolysaccharide) might inhibit T-cell activation from causing immunosuppression. *Brucella* spp. prevent immune activation of macrophages by inducing CD4^+^CD25^+^ T cells to produce the anti-inflammatory cytokine IL-10 during early infection (Xavier et al., 2013). In addition, *B. melitensis* suppresses gamma IFN production and delays memory responses through exhausting CD8^+^T cells (Durward-Diioia et al., 2015). We found that the cattle infected with *Brucella* spp. were susceptible to BoHV-1 and BoHV-4 during our epidemiological investigation. This phenomenon might associate with some component of *Brucella* spp. that could promote the replication of PRV and HSV-1 *via* suppression of the expression of IFN-I. Thus, we investigated the roles of Omp25 in this process, as Omp25 is involved in inhibiting the activity of T cells and macrophages in our previous studies (Cui et al., 2017; Luo et al., 2017).

Cyclic guanosine monophosphate–adenosine monophosphate synthase, a DNA sensor, is essential for IFN-β expression during viral and bacterial infections (Zhang et al., 2014; Collins et al., 2015; Huang Z. F. et al., 2018). Viruses and bacteria have evolved various strategies to prevent activation of the cGAS/STING pathway, resulting in the evasion of host innate immunity. For example, the dengue virus protein NS2B promoting cGAS degradation in autophagy–lysosome-dependent manner results in inhibition of IFN-I production during infection (Aguirre et al., 2017). HSV-1 UL41 was demonstrated to evade the cGAS/STING-mediated DNA-sensing pathway by degrading cGAS mRNA *via* its RNase activity, and HSV-1 VP22 was shown to interact with cGAS and inhibited the enzymatic activity of cGAS (Su and Zheng, 2017; Huang J. et al., 2018; Zheng, 2018). In this study, our results showed that Omp25 that promoted cGAS degradation *via* a ubiquitin–proteasome-dependent pathway led to a decreased cGAMP production in 3D4/21 cells and RAW264.7 cells. Ivashkiv and Donlin (2014) have reported that IRF3 is a master transcriptional factor regulating IFN-I gene induction and innate immune defenses. Some viral proteins can modulate the activation of IRF3 through different processes, including IRF3 dimerization, nuclear localization, and binding to target gene promoters (Schneider et al., 2014). Several HSV-1 proteins, including US3, VP24, VP16, and VZV ORF61, were shown to target IRF3 and inhibit IFN-I production (Zhu et al., 2011; Wang et al., 2013; Xing et al., 2013; Zhang et al., 2016), and PRV UL13 inhibits the IFN-β signaling pathway by targeting IRF3 for ubiquitination and degradation (Lv et al., 2020). This study detected the phosphorylated IRF3 in the cytoplasm and nucleus. The results showed that phosphorylated IRF3 in the nucleus was significantly decreased in Omp25-expressing cells than the cells without Omp25 expression. Thus, we speculate that Omp25 interferes with the translocation of IRF3 to the nucleus, thereby resulting in inhibition for IFN-β transcription.

In summary, the data presented in this work demonstrated that Omp25 promoted the degradation of cGAS by ubiquitin–proteasome to interfere with the activation of the cGAS/STING signaling pathway, to inhibit the production of IFN-β, resulting in the cells with Omp25 expression more easily infected by other pathogens. To the best of our knowledge, this was the first description of Omp25 inhibiting the expression of IFN-β *via* targeting cGAS for degradation. Meanwhile, we cleared the key functional domain of Omp25 that was critical for suppressing the expression of IFN-β. Altogether, our findings presented a novel role of Omp25 in regulating IFN-β production, which might provide novel insights for brucellosis prevention.

## Data Availability Statement

The original contributions presented in the study are included in the article/supplementary material, further inquiries can be directed to the corresponding author/s.

## Author Contributions

RL, WL, YH, and DT conceived and designed the experiments and wrote and revised the manuscript. RL, WL, XY, FZ, ZW, XW, and XZ performed the experiments and analyzed the data. RL, WL, and QD revised the manuscript. All the authors have read and approved the final manuscript.

## Conflict of Interest

The authors declare that the research was conducted in the absence of any commercial or financial relationships that could be construed as a potential conflict of interest.

## Publisher’s Note

All claims expressed in this article are solely those of the authors and do not necessarily represent those of their affiliated organizations, or those of the publisher, the editors and the reviewers. Any product that may be evaluated in this article, or claim that may be made by its manufacturer, is not guaranteed or endorsed by the publisher.
